# Development of a four-item physical activity index from information about subsistence living in rural African women: a descriptive, cross-sectional investigation

**DOI:** 10.1186/1479-5868-6-75

**Published:** 2009-11-13

**Authors:** Ian Cook, Marianne Alberts, Estelle V Lambert

**Affiliations:** 1 Physical Activity Epidemiology Laboratory, University of Limpopo (Turfloop Campus), South Africa; 2 Department of Chemical Pathology, University of Limpopo (Turfloop Campus), South Africa; 3 MRC/UCT Research Unit for Exercise Science and Sports Medicine, University of Cape Town Medical School, South Africa

## Abstract

**Background:**

We investigated the criterion validity of a physical activity index (PAI) derived from socio-demographic variables obtained from convenience samples of rural African women.

**Methods:**

We used a sample (N = 206) from a larger dataset which surveyed adult rural Africans during 1997, and data collected during 2003/4 from 138 adult rural African women. A three-point PAI (low-, medium- and high-subsistence) was constructed from four socio-demographic questions related to electricity, cooking methods, water collection and availability of motorized transport. Criterion measures included measures of adiposity, blood biochemistry, resting blood pressure (RBP), physical fitness (VO_2max_) and single-plane accelerometry (ACC).

**Results:**

Age, educational level and health status were not related to PAI level (p > 0.1). There was a significant negative, linear trend between the PAI level and adiposity level (p < 0.04), and fasting blood glucose concentration (p < 0.0001), while VO_2max _was positively related to PAI level (p = 0.0190). The PAI level was positively and linearly related to ACC output, namely counts.day^-1 ^(p = 0.0044), steps.day^-1 ^(p = 0.0265), min.day^-1 ^of moderate-to-vigorous activity (p = 0.0040), and the percentage of subjects adhering to physical activity public health guidelines (p = 0.0157). Other criterion measures did not reach significance, but were in the expected direction (sedentary behaviour: p > 0.08, RBP: p > 0.07).

**Conclusion:**

The PAI derived from a socio-demographic questionnaire is a valid instrument for broadly categorizing levels of physical activity for this specific population of rural African women. As the epidemiological transition progresses, validity will need to be re-established.

## Background

A host of physical activity questionnaires (PAQ) are available for the quantification of human energy expenditure [1]. PAQ, as an estimate of human energy expenditure, are routinely used to investigate the relationship between physical activity (PA), morbidity and mortality [2]. While the majority of PAQ produce an energy expenditure output e.g. kilojoules, some investigators have used PAQ which consist of a single global question [3] or which produce an index score [4]. The use of physical activity index (PAI) scores has been used with some success in sub-Saharan Africa [5-7]. However, the determination of public health guidelines from an index is problematic. If PA is not the main factor being investigated, but is rather a covariate that must be adjusted for, index scores are quite useful. A case in point is the investigation of the risk of various measures of adiposity in relation to non-communicable diseases such as hypertension, diabetes and dyslipidemia that adjusts for factors such as age, alcohol intake, smoking, energy intake and PA [8]. In order to perform similar analyses on data collected in a rural field site [9] during 1997 [10] for rural adult African females, a valid measure of PA had to be developed because the PAQ used in the original 1997 survey [10] was not tested for validity in rural adult African females. The lack of validity might have displayed a "floor effect" such that important health enhancing PA behaviours were not probed [11]. For example, while vigorous work and leisure PA would be probed, low-to-moderate household and yard PA would be under-reported. Furthermore, any comparison of more recent data with the original survey [10], in terms of PA, would not be possible because the more recent surveys did not include the PAQ originally used. However, more recent surveys have included the same or very similar socio-demographic variables. Because the socio-demographic data contains variables which are directly or indirectly linked to a household's subsistence level, and therefore PA level, we thought it possible to construct and validate a simple PAI. Therefore, this analysis aims to test the criterion validity of a PAI derived from socio-demographic variables, such as availability of motorized transportation, water and electricity supply and cooking methods, such that the PAI can be used as a suitable covariate in multivariate analyses.

## Methods

### Study design

The validation of the PAI consisted of an analysis of two sets of data collected in the Dikgale Health and Demographic and Surveillance System (DHDSS) field site [9,12]. Part 1 of the analysis used data collected in 1997, which has been reported in detail elsewhere [10], and included anthropometry, blood biochemistry, resting systolic and diastolic blood pressure (RBP) and socio-demographic information. Part 2 of the analysis used data collected in 2003/4 and included anthropometry, objectively measured PA, physical fitness (PF), RBP and socio-demographic information. Both studies obtained signed informed consent from all participants, and were approved by the Ethics Committee of the University of Limpopo.

### Subjects

#### Part 1: 1997 data

For this analysis, a subset of 206 adult (30 to 55 years), non-pregnant, rural African women, with complete data for variables relating to socio-demographics, anthropometry, blood biochemistry and RBP was selected from an existing dataset [10].

#### Part 2: 2003/4 data

Data from a convenience sample of 138 adult (19 to 56 years), non-pregnant, rural African women, permanently resident in the DHDSS site, were included in this analysis. The participants were contacted twice over an eight-day period. On the first occasion, subjects were recruited and completed the informed consent and a socio-demographic questionnaire. All interviews were conducted by trained, local female fieldworkers. After 10 minutes of seated rest, RBP was recorded. Thereafter anthropometric and PF measures were obtained. Trained female medical students conducted the anthropometric, RBP, and PF measurements. Finally, subjects were instructed on the required procedures for wearing the accelerometer. Eight days later the accelerometers were collected. Subjects received a small honorarium on completion of the study.

### Anthropometric measurements

#### Part 1 and 2: 1997 and 2003/4 data

Standard anthropometric measures of stature (nearest 0.1 cm), body mass (nearest 0.1 kg) and waist circumference (WC, nearest 1 mm) were taken in duplicate with the subjects in light clothing and without shoes, and the average calculated. Additionally, in the 2003/4 sample, skinfold thicknesses (triceps, biceps, subscapular, suprailiac; nearest 1 mm, average of triplicate measurement) were measured according to standardised procedures [13]. From these measures body mass index (BMI = body mass ÷ stature^2 ^= kg.m^-2^) and sum of skinfolds (SSKF = triceps + biceps + subscapular + suprailiac, mm) were calculated.

### Biochemistry

#### Part 1: 1997 data

A registered nurse collected fasting blood samples. After having been seated for at least 15 min, 30 ml blood was drawn from each subject into either plain tubes or tubes containing ethylenediamine tetra-acetic acid or sodium fluoride. The blood samples were stored in a coolbox and then centrifuged within 5 h to prepare the plasma or serum. Aliquots were frozen at -70°C until analysed. The sodium fluoride plasma was used for measuring glucose and was analysed on a Technicon RA-XT, using reagents from Bayer Diagnostica, New York, USA. The remaining components were measured using Dimension ES and reagents from Dade Behring, USA. Low-density lipoprotein cholesterol (LDL-C) was calculated using the Friedewald equation [14]. The inter-run coefficient of variation for glucose (GLUC), total cholesterol (TC), high-density lipoprotein cholesterol (HDL-C), and triglyceride (TG) was 3.6%, 2.2%, 1.4% and 4.2%, respectively. The intra-run coefficient of variation for GLUC, TC, HDL-C, and TG was 4.2%, 2.3%, 1.8% and 3.1%, respectively.

### Resting blood pressure

#### Part 1 and 2: 1997 and 2003/4 data

RBP (mmHg) was recorded with a mercury sphygmomanometer [10] (Part 1: 1997 data) and a validated automated blood pressure monitor [15] (Part 2: 2003/4 data). Three readings were taken, and the average of the last two readings was used as a measure of RBP.

### Physical Fitness Level

#### Part 2: 2003/4

Maximal oxygen uptake (VO_2max_) was estimated using the submaximal, Siconolfi step test [16] which has been shown to correlate well with measured VO_2max _(r = 0.86 to 0.92) [16,17] and has been used in other indigenous populations [18]. Briefly, the participants stepped on a 25.4 cm high bench for three minutes per stage for a maximum of three stages, or until their exercise heart rate for a stage exceeded 70% of age-predicted maximum heart rate (220 - age). Heart rate was measured using a heart rate monitor (Polar XTrainerPlus, Polar Electro OY, Finland). Exclusion criteria included a medical history of cardiovascular, respiratory, or severe muscular-skeletal disease or an unwillingness to perform the test. Trained female medical students supervised the testing.

### Accelerometry

#### Part 2: 2003/4 data

To objectively quantify the PA of the subjects, single-plane accelerometers were worn for approximately seven days (mean: 6.95 days, minimum: 6.47 days, maximum: 7.50 days). The mean time for which counts were recorded (counts.min^-1 ^> 0) was 9.3 hrs.day^-1 ^(minimum: 5.4 hrs.day^-1^, maximum: 12.9 hrs.day^-1^). The MTI Actigraph accelerometer (model AM-7164-2.2, MTI Health Services, Fort Walton Beach, FL) is small and unobtrusive (5 cm × 4 cm × 1.5 cm, 43 g) and has been described in detail elsewhere [19]. In this study, the epoch duration was set at one minute and the pedometer mode was activated. The accelerometer was worn on the right waist, securely attached to a nylon belt. The accelerometers could be removed for sleeping and bathing purposes by unclipping the nylon belt. The data was downloaded from the accelerometers onto an IBM-compatible personal computer via an interface unit, for further analysis. From at least five complete recording days, an average PA volume was defined as steps.day^-1 ^and counts.day^-1^, and PA intensity patterns as min.day^-1 ^of sedentary, light and moderate-to-vigorous activity. The cut-points for moderate (3-6 METs, 1 MET = 1 Metabolic Equivalent = 3.5 mlO_2_.kg^-1^.min^-1 ^= 1 kcal.kg^-1^.hr^-1^) and vigorous (>6 METs) activity was 574 counts.min^-1 ^and 4945 counts.min^-1^, respectively [20]. Sedentary behaviour was defined as counts.min^-1 ^= 0, and included lying, sitting and standing still. Light activity was defined as counts.min^-1 ^> 0 but less than 574. From the accelerometry data we determined compliance with two PA public health guidelines. The American College of Sports Medicine/Centres for Disease Control (ACSM/CDC) guidelines recommend moderate-to-vigorous PA of 30 minutes.day^-1^, at least 5 days.week^-1^, accumulated in bouts of ≥10 minutes [21]. The Institute of Medicine (IOM) guidelines recommend moderate-to-vigorous PA of 60 minutes.day^-1^, at least 5 days.week^-1^, accumulated in bouts of ≥10 minutes [22].

### Physical Activity Index

The PAI was derived from four questions, found within a socio-demographic questionnaire which was used in the original survey conducted in 1997 [10] and the more recent 2003/4 sample. The questions probed availability of electricity in the dwelling, collection of water, use of wood for cooking, and access to a motor vehicle within the household.

The rationale for using these particular questions was that within this particular community, at present, much of the PA performed by women revolves around housework and yard work. Communal lands are available for planting of crops, such as maize and some vegetables, and cattle grazing. The lack of electricity in the dwelling precludes the use of labour saving devices such as washing machines and vacuum cleaners, while a supply of electricity could encourage greater sedentary activity due to electronic devices such as television [23,24]. The use of wood for cooking is still common and requires the collection (saws, axes) and transport (head panning, wheelbarrows) of the raw material or purchasing of wood from sellers coming to the villages. If water is not available in or around the dwelling, water is collected in containers and transported principally through head panning or wheelbarrows. Journeys to the local store, clinic, communal fields, and to bus or taxi stops are completed on foot, if there is no access to motor vehicle transport within the household [25]. Leisure activities prevalent in urban settings are not encountered in the DHDSS.

Consequently, we felt that these four questions provided a reasonable categorical estimate of the most common activities performed by the majority of women in the DHDSS. The questions, possible responses and scoring are set out in Table 1. A score of 1 was allocated to the option which would more likely result in greater energy expenditure. The lowest and highest scores possible are zero and four, respectively, which we translated into three arbitrary categories; low subsistence level (total score ≤2, low activity level), medium subsistence level (total score = 3, medium activity level) and high subsistence level (total score = 4, high activity level).

**Table 1:** The questions, responses and scoring of the derived Physical Activity Index

Question and Responses	Scoring
Do you have electricity in the house (Yes/No)	Yes = 0, No = 1
Do you use wood for cooking? (Yes/No)	Yes = 1, No = 0
Does any person in your household own a motor vehicle? (Yes/No)	Yes = 0, No = 1
How is your water supplied? (Select one)	
Tap inside the house	Yes = 0
Tap outside the house	Yes = 0
Shared tap	Yes = 1
Communal tap	Yes = 1
Water truck	Yes = 1
Total score =	Sum of responses

### Statistical analysis

To test the validity of our PAI, the trend (linear and non-linear) across the three PAI categories was tested for continuous measures (adiposity, blood biochemistry, RBP, PA volumes and intensity patterns), and frequencies (adherence to PA public health guidelines). One-way Analysis of Variance and Univariate General Linear Model analyses (age and BMI or WC as covariates where required) were used for continuous variables across PAI categories. Independent t-tests examined possible differences between the two datasets. For categorical data, a Chi-square test for trend was used across PAI categories, and independent tests of proportions examined possible differences between the two data sets [26]. We expected inverse trends for measures of adiposity, GLUC, TC, TG, LDL-C, RBP, sedentary activity, and light activity with increasing PAI index. HDL-C, PF, PA volumes, moderate-to-vigorous activity, and adherence to public health guidelines were expected to increase with increasing PAI index. For comparative purposes we also performed correlational analyses (Kendall's τ) between the PAI index and continuous criterion measures, namely adiposity, PF, GLUC, TG, TC, LDL-C, HDL-C, PA volumes, sedentary activity, light activity, moderate-to-vigorous activity, and adherence to public health guidelines. Thereafter, Kendall's τ was converted to Pearson's *r *[27]. First-order partial correlation coefficients were calculated as required. Where necessary continuous data were transformed (log_e_) and expressed as a geometric mean. Data were analysed using appropriate statistical software (SPSS Inc. SPSS Statistical Software: Release 17.0.0 SPSS Corp, Chicago, Il, 2008). Significance for all inferential statistics was set at p < 0.05, and 95%CI were constructed for means and proportions.

## Results

Subject characteristics for both parts of the analysis are reported in Table 2. Not all the subjects completed the fitness test due to uncontrolled hypertension, epilepsy or personal reasons (n = 7). Noticeable are the significant increases in electricity supply (53%, p = 0.0002) within dwellings and educational achievement (31.4%, p < 0.0001) from 1997 to 2003/4 (Table 2). PAI scores tended to be evenly distributed across subsistence level categories for the 1997 data, but tended to aggregate toward the low-to-medium subsistence level categories for the 2003/4 data. There was a significant decrease in the high subsistence PAI level category from 1997 to 2003/4 (p < 0.05) (Table 2).

**Table 2:** Subject physical and socio-demographic characteristics

	***Part 1: 1997 data*** ***(N = 206)***	***Part 2: 2003/4 data*** ***(N = 138)***
	
**Health and Socio-demographic^1^**		
Diagnosed with hypertension and/or diabetes	31.4 (25.2 to 38.3)	21.0 (15.0 to 28.6) ^6^
Prevalence of body mass index ≥30 kg.m^-2^	30.6 (24.7 to 37.2)	33.3 (26.0 to 41.6)
Prevalence of waist circumference ≥88 cm	36.4 (30.1 to 43.2)	35.5 (28.0 to 43.8)
Achieved educational level of Gr. 8	23.7 (18.3 to 30.2)	55.1 (46.7 to 63.1) ^6^
Electricity inside house	34.0 (28.8 to 39.6)	87.0 (80.3 to 91.6) ^6^
Wood used for cooking purposes	83.3 (78.8 to 87.3)	89.9 (83.7 to 93.9)
One or more persons owns a motor vehicle	18.9 (14.8 to 23.8)	16.7 (11.4 to 23.8)
Water supplied by tap in or around dwelling	52.0 (46.2 to 57.7)	56.5 (48.2 to 64.5)
**Physical Activity Index^1^**		
Low subsistence level (score ≤2, low activity level)	39.3 (32.9 to 46.1)	19.6 (13.8 to 27.0) ^6^
Medium subsistence level (score = 3, medium activity level)	25.2 (19.8 to 31.6)	57.3 (48.9 to 65.2)^6^
High subsistence level (score = 4, high activity level)	35.4 (29.2 to 42.2)	23.2 (16.9 to 30.9) ^6^
**Anthropometry and fitness**		
Age (years)	43.6 (42.6 to 44.6)	36.3 (34.6 to 37.9) ^6^
Body mass index (kg.m^-2^)	26.8 (25.9 to 27.6)	26.9 (25.8 to 27.9)
Waist circumference (cm)	83 (82 to 85)	83 (81 to 85)
Sum of four skinfolds (mm) ^2^	-	65 (59 to 71)
VO_2max _(mlO_2_.kg^-1^.min^-1^) ^3^	-	26.7 (25.6 to 27.7)
**Blood biochemistry**		
Blood glucose (mmol.l^-1^) ^2^	5.20 (4.95 to 5.45)	-
Triglyceride (mmol.l^-1^) ^2^	1.05 (0.90 to 1.01)	-
Total cholesterol (mmol.l^-1^)	4.52 (4.37 to 4.67)	-
HDL-cholesterol (mmol.l^-1^)	1.19 (1.16 to 1.21)	-
LDL-cholesterol (mmol.l^-1^) ^2^	2.69 (2.56 to 2.82)	-
**Resting blood pressure**		
Systolic blood pressure (mmHg)	129 (126 to 132)	120 (117 to 122) ^4^
Diastolic blood pressure (mmHg)	84 (83 to 86)	77 (75 to 79) ^4^
**Accelerometry**		
Counts.day^-1 ^(× 1000)	-	411 (390 to 432)
Steps.day^-1 ^(× 1000)	-	13.5 (13.0 to 14.1)
Sedentary activity (min.day^-1^)	-	797 (780 to 814)
Light activity (min.day^-1^)	-	390 (375 to 405)
Moderate-to-vigorous activity (min.day^-1^)	-	253 (241 to 266)
**Adherence to public health guidelines **^1^		
ACSM/CDC Guidelines ^4^	-	96.4 (91.8 to 98.4)
IOM Guidelines ^5^	-	69.6 (61.4 to 76.6)

Values are reported as mean (95%CI) except ^1 ^% (95%CI) and ^2 ^geometric mean (95%CI); ^3 ^n = 131; ^4 ^ACSM/CDC guidelines: moderate-to-vigorous PA of 30 minutes.day^-1^, at least 5 days.week^-1^, accumulated in bouts of ≥10 minutes; ^5 ^IOM guidelines: moderate-to-vigorous PA of 60 minutes.day^-1^, at least 5 days.week^-1^, accumulated in bouts of ≥10 minutes; ^6 ^significant differences between the two datasets, p ≤ 0.05

### Part 1: 1997 data

We found no statistically significant linear trend between PAI level and age (p = 0.8852) or educational achievement of at least Gr. 8 (Grade 8: first year of secondary or high school, p = 0.0516). Self reported diagnosis with, or on medication for, hypertension and/or diabetes was linearly related to PAI level (p = 0.0180). However, using objective measures of RBP and GLUC levels found no linear trend between hypertensive (≥140/90 mmHg) [10] and/or diabetic (fasting GLUC ≥7.0 mmol.l^-1^) states [10] and PAI level (p = 0.4093). Age did not differ significantly between any of the PAI levels (p = 0.2121). The trend analysis for age-adjusted continuous criterion variables is reported in Table 3. Significant age-adjusted linear decreases in BMI (p = 0.0091), WC (p = 0.0003) and GLUC (p < 0.0001) with increasing PAI were found, while the age-adjusted linear trend for LDL-C did not quite reach statistical significance (p = 0.0650). The linear trend for GLUC remained significant after adjusting for age and BMI or age and WC (p < 0.0001). Generally, other criterion variables were relatively constant across PAI levels. Non-linear trends for continuous variables across PAI categories were not significant (p > 0.2). Correlational analysis between the PAI score and continuous criterion variables mirrored the findings of the linear analyses. Significant coefficients were obtained for BMI (r = -0.24, p = 0.0037), WC (r = -0.30, p = 0.0004) and GLUC (r = -0.47, p < 0.0001). The association between PAI and GLUC remained significant, even after adjusting for BMI (r = 0.20, p = 0.0034). Age was not significantly related to either GLUC (r = 0.09, p = 0.2105) or the PAI score (r = -0.03, *p *= 0.6893).

**Table 3:** Part 1 (1997 dataset): Linear trend across three PAI categories for continuous variables

	**Subsistence and Physical Activity level**			
	
	**Low (PAI score ≤2)** **b(n = 81)**	**Medium (PAI score = 3)** **(n = 52)**	**High (PAI score = 4)** **(n = 73)**	**Significance level**
	
**Anthropometry and fitness**				
Body mass index (kg.m^-2^)	28.1 (26.8 to 29.5)	26.4 (24.7 to 28.1)	25.5 (24.1 to 27.0)	0.0091 ^2^
Waist circumference (cm)	87 (85 to 90)	82 (79 to 86)	80 (77 to 83)	0.0003 ^2^
**Resting blood pressure**				
Systolic blood pressure (mmHg)	132 (128 to 137)	128 (123 to 134)	126 (122 to 131)	0.0754
Diastolic blood pressure (mmHg)	85 (82 to 87)	84 (81 to 88)	84 (82 to 87)	0.7305
**Blood biochemistry**				
Blood glucose (mmol.l^-1^) ^1^	5.96 (5.54 to 6.41)	4.95 (4.52 to 5.42)	4.62 (4.28 to 4.99)	< 0.0001 ^2^
Triglyceride (mmol.l^-1^) ^1^	0.96 (0.88 to 1.05)	0.96 (0.86 to 1.07)	0.94 (0.86 to 1.03)	0.7668
Total cholesterol (mmol.l^-1^)	4.67 (4.44 to 4.90)	4.41 (4.12 to 4.70)	4.43 (4.19 to 4.68)	0.1631
HDL-cholesterol (mmol.l^-1^)	1.19 (1.15 to 1.22)	1.18 (1.13 to 1.23)	1.20 (1.15 to 1.24)	0.7563
LDL-cholesterol (mmol.l^-1^) ^1^	2.85 (2.64 to 3.08)	2.60 (2.36 to 2.86)	2.57 (2.37 to 2.79)	0.0650

Values are reported as age-adjusted mean (95%CI) except ^1 ^age-adjusted geometric mean (95%CI); ^2 ^significant linear trend for low-to-high active

### Part 2: 2003/4 data

We found no linear trend between PAI level and age (p = 0.4919), educational achievement of at least Gr. 8 (p = 0.1644), or diagnosis with or on medication for hypertension and/or diabetes (p = 0.6741). Age did not differ significantly between any of the PAI groups (p = 0.1292). The trend analysis for continuous (age adjusted) and categorical criterion variables is reported in Table 4. After adjustment for age, SSKF decreased significantly with increasing PAI level (p = 0.0322) and VO_2max _increased significantly with increasing PAI (p = 0.0190). Other measures of adiposity tended to decrease with greater PAI level but not significantly so (p > 0.08). PA volumes (counts.day^-1 ^and steps.day^-1^), moderate-to-vigorous activity levels and adherence to IOM Guidelines showed significant linear trends, and in the expected direction (p < 0.03). Sedentary behaviour was inversely related to PAI, but did not quite reach statistical significance (p = 0.0806). Non-linear trends for continuous variables across PAI categories were not significant (p ≥ 0.2). Correlational analysis between the PAI score and continuous criterion variables mirrored the findings of the linear analyses. Significant coefficients were obtained for VO_2max _(r = 0.21, p = 0.0466), counts.day^-1 ^(r = 0.26, p = 0.0113), steps.day^-1 ^(r = 0.23, p = 0.0292) and moderate-to-vigorous activity (min.day^-1^) (r = 0.27, p = 0.0085). Age was not significantly related to the PAI score (r = 0.06, *p *= 0.5761).

**Table 4:** Part 2 (2003/4 dataset): Linear trend across three PAI categories for continuous and categorical variables

	**Subsistence and Physical Activity level**			
	
	**Low (PAI score ≤2)** **(n = 27)**	**Medium (PAI score = 3)** **(n = 79)**	**High (PAI score = 4)** **(n = 32)**	**Significance level**
	
**Anthropometry and fitness**				
Body mass index (kg.m^-2^)	28.1 (25.8 to 30.4)	27.1 (25.8 to 28.4)	25.2 (23.1 to 27.3)	0.0617
Waist circumference (cm)	86 (81 to 91)	84 (81 to 87)	80 (75 to 84)	0.0703
Sum of four skinfolds (mm) ^1^	74 (60 to 90)	67 (59 to 75)	55 (45 to 66)	0.0322 ^8^
VO_2max _(mlO_2_.kg^-1^.min^-1^)	25.7 (23.8 to 27.5) ^5^	26.2 (25.1 to 27.3) ^6^	28.7 (27.0 to 30.5) ^7^	0.0190 ^8^
**Resting blood pressure**				
Systolic blood pressure (mmHg)	119 (114 to 125)	120 (116 to 123)	120 (115 to 125)	0.8387
Diastolic blood pressure (mmHg)	78 (74 to 82)	77 (74 to 79)	78 (74 to 81)	0.9611
**Accelerometry**				
Counts.day^-1 ^(× 1000)	364 (317 to 411)	409 (381 to 436)	457 (414 to 501)	0.0044 ^8^
Steps.day^-1 ^(× 1000)	12.3 (11.0 to 13.6)	13.6 (12.9 to 14.4)	14.3 (13.1 to 15.5)	0.0265 ^8^
Sedentary activity (min.day^-1^)	828 (789 to 866)	793 (770 to 815)	781 (746 to 817)	0.0806
Light activity (min.day^-1^)	387 (354 to 420)	396 (377 to 416)	377 (346 to 408)	0.6755
Moderate-to-vigorous activity (min.day^-1^)	225 (198 to 253)	251 (235 to 267)	282 (256 to 307)	0.0040 ^8^
**Adherence to public health guidelines **^2^				
ACSM/CDC Guidelines ^3^	96.0 (80.5 to 99.3)	98.5 (91.8 to 99.7)	93.8 (83.2 to 97.9)	0.5678
IOM Guidelines ^4^	51.9 (34.0 to 69.3)	70.9 (60.1 to 79.8)	81.3 (64.7 to 91.1)	0.0157 ^8^

Values are reported as age-adjusted mean (95%CI) except for ^1 ^age-adjusted geometric mean (95%CI) and ^2 ^% (95%CI); ^3 ^ACSM/CDC guidelines: moderate-to-vigorous PA of 30 minutes.day^-1^, at least 5 days.week^-1^, accumulated in bouts of ≥10 minutes; ^4 ^IOM guidelines: moderate-to-vigorous PA of 60 minutes.day^-1^, at least 5 days.week^-1^, accumulated in bouts of ≥10 minutes; ^5 ^n = 26; ^5 ^n = 75; ^7 ^n = 30; ^8 ^significant linear trend for low-to-high active

## Discussion

The principal finding of this analysis was that a three-point PAI derived from four questions within a socio-demographic questionnaire, was significantly associated with a number of criterion measures namely adiposity, fasting blood glucose, VO_2max _and raw and derived-accelerometry output. Furthermore, the relationship of a number of other criterion variables with the PAI was in the expected direction, but did not reach statistical significance. These results suggest that the derived PAI is valid for broadly categorizing levels of PA for this particular population, at this particular time. Importantly, this analysis was novel because a PA measure had to be constructed and validated from socio-demographic data collected in 1997 and 2003/4, so that the PAI could be used as a covariate in an analysis of data collected in 1997 [10]. In other words, the validation analysis was performed on two datasets separated by six years.

The variables we chose to comprise the PAI affect the possession or not of a number of assets which in turn directly or indirectly decrease or increase PA behaviour and hence energy expenditure levels. These variables do not narrowly confine their influence within a household to a small number of tasks which might affect only a few members of the household. Rather, the effect is fundamental and pervasive such that the entire household is affected in terms of PA choices and behaviours and consequent energy expenditure levels [23-25]. Moreover, our experience has been that in agrarian households, workload is spread relatively evenly throughout the female population, unless very ill or incapacitated. The chores might not be all equal in nature, but rather that the general activity level is higher in that household, especially so for women. These subsistence tasks performed by the household are usually light-to-moderate in intensity, spread out over a large portion of the day which is in contrast to Western productivity which requires high bursts of energy over relatively shorter periods of time [28]. Moreover, much of the decline in PA in industrialised settings has been through the reduction in subsistence PA, namely occupation, household and yard PA [29]. The reduced subsistence PA, and resultant positive energy balance, in highly industrialised, urbanised settings has led to a concomitant rise in obesity and various chronic diseases of lifestyle of epidemic proportions [29].

The results highlight the relatively early stage of epidemiological change in which this community finds itself. The implication is that the PAI would not be valid in years to come as PA behaviours shift to more sedentary choices and patterns [30]. Investigations in developing economies have found a wide range of PA levels based on occupation and gender [31,32], which is not the case in industrialised countries where leisure-time pursuits tend to dominate PA choices [33]. Because criterion validity was established for 1997 and 2003/4 datasets, it would seem that PA, driven by subsistence demands, have not altered markedly over this six year period.

The changes in electricity supply and educational achievement are probably because of the extensive electrification projects within rural communities by the national electricity supplier and the national imperative to extend education within all communities in child and adult populations. The significantly lower diabetes and/or hypertension prevalence for the 2003/4 sample compared with the 1997 sample is likely due to the convenience sampling and selection criteria, and also because the 2003/4 sample is significantly younger than the 1997 sample. RBP is significantly higher in the 1997 sample compared with the 2003/4 sample and is probably due to sampling methods and selection criteria, the subject age differential between the two datasets, and because different instruments were used to measure RBP.

Our results are in accord with other work which has shown that rural women carry a relatively heavy work burden, particularly with respect to household and yard work, and aspects of farming [34]. Likewise, Walker *et al*. have noted the physically active lifestyle of rural African women [35]. Surprisingly, similar PA patterns have been reported for urban African women in an informal settlement [36]. It is plausible that the PA levels of these women is comparable to that of rural residents, considering that informal settlements often lack modern amenities, much like rural settings [36]. Paradoxically, some studies have found deep rural African women to be less physically active than women resident on farms or in informal settlements [31]. Generally, these studies would seem to suggest that our choice of questions provides a fairly robust, albeit crude, measure of the PA demands placed on rural African women. Indeed, our findings bear out the observation of Baranowski as to the "robustness of the phenomenon" with regards to the quantification of PA [37]. This does not imply that researchers need not rigorously validate PA instruments for specific populations, but rather that meticulously investigated validity, particularly content validity, invariably results in robust measures of PA. Although we did not test reliability, we would argue that the questions are logical, unambiguous, easily understood within any ethnic or language context, and that the aspects which the questions probe are stable over time, such that reliability would be expected to be high. In this regard Kruger *et al. *found an intra-class correlation coefficient of 0.88 with a more extensive PAI administered to rural and urban subjects [4]. However, further reliability studies would need to be conducted to test our assertion.

The use of PA indexes in other studies in sub-Saharan Africa have shown similar trends, albeit that none of these studies used trend analysis across ordered groups, which is the recommended statistical technique for such data [26]. Generally, these studies have used analysis of variance with *post hoc *analysis, and correlation analysis. Morrison *et al. *found significant associations between an eight-point PA index probing leisure-time PA and measures of fitness and adiposity in a sample of 1015 Caucasian Zimbabweans [6]. The retrospective analysis by Sparling *et al. *used data obtained from 212 urban African males [5]. The PA index probed within-work and after-work PA categories. Both categories consisted of three groupings (low-to-high). The trend between the PA index and measures of adiposity, blood pressure and serum lipids was not as pronounced as might be expected. In fact, the most favourable blood pressure and serum lipid profiles were found within low-to-moderate PA levels. The data of Sparling *et al. *reveals no significant linear trends in within-work PA for measures of adiposity, blood pressure or serum lipids (p ≥ 0.1495) [5]. However, for age-adjusted data, the trends were stronger for after-work PA, but did not reach significance (p ≥ 0.0782) [5]. Kruger *et al. *investigated the validity and reliability of a more extensive PA index in 206 rural and urban Africans [4]. Significant associations were found between the PA index score and measures of adiposity. Furthermore, adiposity in the most active quartile was significantly lower than the least active quartile. Similarly, energy intake was significantly higher in the most active quartile. Results provided in the Kruger *et al. *paper allowed us to determine that significant linear trends (p ≥ 0.0006) exist for measures of adiposity and energy intake across the four quartiles of activity [4].

Although linear trend analysis across ordered groups is the recommended statistical technique for such data [26] we have reported correlation coefficients for comparative purposes. PAI criterion validity correlation coefficients (median: r = 0.24, range: r = 0.20 to r = 0.30), fell within the range of values reported for a number of self report physical activity questionnaires (r = 0.14 to r = 0.53) [38] but did not reach the range of values reported for the recently validated International Physical Activity Questionnaire [39] and the WHO Global Physical Activity Questionnaire [40]; (r = 0.31 to 0.52). Considering the length and detailed nature of the comparative questionnaires [38-40], the PAI criterion validity correlation coefficients were remarkably large.

The strengths of the study were firstly that novel data was reported from the sub-Saharan African region, specifically for accelerometry and physical fitness, in an under-reported segment of the population, namely rural African women. Secondly, the study highlights the fact that the workload of rural African women is influenced to a large extent by economic and developmental variables. Thirdly, significant associations were found in both datasets, using a variety of indirect and direct criterion variables. Lastly, despite the constraints imposed by the *post hoc *development and testing of the PAI, the criterion validity coefficients were reasonable when compared with results from other validation studies.

Weaknesses of the study were firstly the limitations imposed by the datasets in terms of available variables, such that we could not conduct more in-depth analyses on other factors that might affect the workload for household and individual female members of the household. We would thus caution against the use of this particular PAI score as a general measure of PA in rural African women without further testing and refinement to address more fully aspects of internal and external validity. Secondly, we could not address the energy intake levels across the PAI subsistence levels, and thus energy balance with the concomitant influences on anthropometric indices and blood biochemistry. Lastly, although we did compare results between the two datasets, the comparison is limited because both samples were convenience samples selected according to different criteria.

## Conclusion

This analysis suggests that a simple PAI, derived from socio-demographic variables, is a valid but probably temporary measure of PA within a specific population of rural African women, and can be included as a covariate in multivariate analyses using a 1997 dataset.

## Competing interests

The authors declare that they have no competing interests.

## Authors' contributions

IC was involved in the conception and design of the research study, did the data cleaning and analysis, and was responsible for the drafting and manuscript writing. MA was involved in the conception and design of the research study and played a role in critically revising and editing the manuscript, and is project leader of the Dikgale Health and Demographic Surveillance System Site. EVL was involved in the conception and design of the research study and played a role in critically revising and editing the manuscript. All authors have read and approved the final manuscript.
